# Modulation of the relationship between spring AO and the subsequent winter ENSO by the preceding November AO

**DOI:** 10.1038/s41598-018-25303-0

**Published:** 2018-05-02

**Authors:** Shangfeng Chen, Wen Chen, Bin Yu

**Affiliations:** 1 Center for Monsoon System Research, Institute of Atmospheric Physics, Chinese Academy of Sciences, Beijing, China; 2 Climate Research Division, Environment and Climate Change Canada, Toronto, ON Canada

## Abstract

Previous studies indicated that the spring Arctic Oscillation (AO) exerts significant influences on the subsequent winter El Niño-Southern Oscillation (ENSO). This analysis suggests that the spring AO-ENSO linkage is highly modulated by its preceding November AO. When November and the subsequent spring AO indices are in phase, the spring AO has a pronounced influence on ENSO. However, when the November and spring AO indices are out of phase, the spring AO-ENSO connection disappears. Modulation of the November AO on the spring AO-ENSO connection is mainly through the constructive and destructive superposition of the November and spring AO associated sea surface temperature (SST) anomalies in the tropical central-eastern Pacific in spring and summer, as well as the SST anomalies developed further in the tropical Pacific via the positive air-sea feedback.

## Introduction

The El Niño–Southern Oscillation (ENSO) is the strongest interannual ocean-atmospheric coupled mode in the tropics and has notable impacts on weather and climate variations over many parts of the globe^1–8^. Thus understanding the driving factor of ENSO variability has been a long-standing hotspot issue. Besides the well-known air-sea interaction and oceanic dynamical processes within the tropical Pacific, studies indicated that atmospheric forcings over the extratropics are also important in forming and maintaining the ENSO variability^9–15^.

Regarding the extratropical atmospheric forcing, recent studies suggested that the Arctic Oscillation (AO), the leading mode of atmospheric variability over the Northern Hemisphere^16^, in spring could exert significant impacts on the subsequent winter ENSO via inducing anomalous westerly winds over the tropical western-central Pacific (TWCP)^12,13,15^. Specifically, when spring AO is in its positive phase, significant westerly wind anomalies are observed over the TWCP. These TWCP westerly wind anomalies result in SST warming anomalies in the tropical central-eastern Pacific (TCEP) in the following summer via triggering an eastward propagating warm Kelvin wave. The SST warming in the TCEP maintains and develops subsequently into an El Niño event in the following winter via the positive air-sea feedback mechanism^1,15^.

In addition to the spring AO influence, Chen *et al*.^17^ found that November AO also affects the SST anomalies in the TECP in the following spring and summer. However, the influence of the November AO on the TECP SST variation in the subsequent winter one year later, which is usually related to an ENSO event, is weak^17^. Nevertheless, the November AO-induced SST anomalies may perturb the spring AO-induced SST anomalies in the TECP in the following summer, which may further impact the formation of El Niño (La Niña)-like SST anomalies in the following winter via enhancing (suppressing) the positive air-sea interaction over the tropical Pacific. Hence, it is speculated that the preceding November AO may modulate the spring AO-ENSO relationship. Evidence to support this modulation hypothesis is provided in this study.

## Data and Methods

Monthly mean atmospheric variables were provided by the European Centre for Medium-Range Weather Forecasts (ERA) Interim Reanalysis from 1979 to the present^18^. Monthly mean SST data were obtained from the National Oceanic and Atmospheric Administration (NOAA) Extended Reconstructed SST version 3b (ERSSTv3b) from 1854 to the present^19^. Monthly mean precipitation data were derived from the Global Precipitation Climatology Project (GPCP) from 1979 to the present^20^. In addition, the monthly mean AO index was extracted from the Climate Prediction Center of NOAA dataset (http://www.cpc.ncep.noaa.gov). The analyzed time period in this study is 1979–2015 during which all the variables are available.

Following the previous study^17^, the preceding November Niño3.4 index (area-averaged SST anomalies over 5°S–5°N and 170°–120°W) has been linearly removed from the AO index and all the analyzed variables. This intends to ensure that results obtained are not influenced by the ENSO cycle. In addition, all variables of interest are subjected to an 11-yr high-pass Lanczos filter^21^ since we focus on interannual variations. Significances of correlation and regression coefficients are estimated based on the two tailed Student’s *t* test.

## Results

The correlation coefficient between the spring AO index and the following winter Niño3.4 index reaches 0.4 over 1979–2014 (Supp. Fig. S1), significant at the 95% confidence level, consistent with previous findings of the relationship between the spring AO and the subsequent winter ENSO^12,15^. The physical process of the spring AO-ENSO relationship can be summarized briefly as follows: in positive spring AO years, a pronounced atmospheric dipole anomaly pattern occurs over the North Pacific, with an anomalous anticyclone and cyclone over the mid-latitudes and the subtropics of North Pacific, respectively (Supp. Fig. S2a). Formation of the cyclonic anomaly in the subtropics is attributed to the interaction between synoptic-scale eddies and the low frequency mean flow and its associated vorticity transportation^15^. The anomalous cyclone associated southwesterly wind anomalies over the subtropical western-central Pacific reduce the climatological northeasterly wind, leading to SST warming there via a reduction of the evaporation and upward latent heat flux^15,22,23^. Meanwhile, enhancement of the anomalous atmospheric heating over the subtropical western-central Pacific related to the SST warming plays an important role in the generation and maintenance of the westerly wind anomalies over the TWCP via a Gill type atmospheric response^15,24^ (Supp. Figs S2a and S3a). The westerly wind anomalies over the TWCP extend eastward, leading to positive SST anomalies over the TCEP in the following summer by triggering eastward propagating and downwelling Kelvin waves^12,25–27^ (Supp. Fig. S2a,b). Finally, the positive SST and associated atmospheric heating and atmospheric circulation anomalies in the tropical Pacific would sustain and develop into an El Niño event via the positive Bjerknes air-sea feedback mechanism^1,15^ (Supp. Figs S2b–d and S3b–d).

Besides the influence of the AO on the tropical Pacific SST, it is noted that SST anomalies in the tropical Pacific associated with the ENSO may also exert impacts on the AO-like atmospheric circulation^28–30^. In addition, it is well known that ENSO has a strong autocorrelation (i.e., quasi-biennial variability). This brings a question here: whether the significant connection between the spring AO and the following winter ENSO identified in previous studies^12,15^ were induced by the ENSO cycle? To address this issue, we remove the D(−1)JF(0) Niño3.4 index from the MA(0) AO index and D(0)JF(1) Niño3.4 index before the calculation. We then recalculate the seasonal evolutions of the SST and atmospheric circulation anomalies regressed on the MA(0) index. The results are shown in Supp. Fig. S4. It is found that the evolutions of the atmospheric circulation and SST anomalies in Supp. Fig. S4 closely resemble those shown in Supp. Fig. S2. In particular, El Niño-like SST warming is still clearly observed in the tropical central-eastern Pacific during the following winter related to the positive spring AO index. Hence, the significant spring AO-winter ENSO connection obtained from the previous studies^12,15^ tends to be independent of the ENSO cycle.

**Figure 1:**
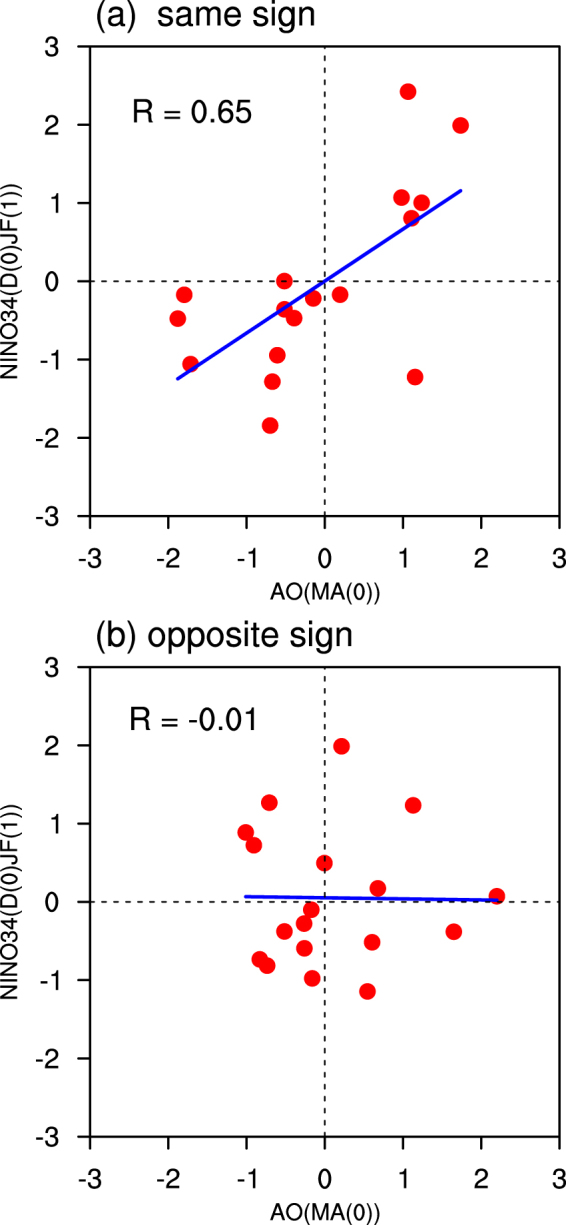
Scatterplots of the spring AO index with the subsequent winter Niño3.4 index, for the years when the spring AO index and the preceding November AO index have the (**a**) same and (**b**) opposite signs, respectively.

Next, we examine the influence of November AO on the subsequent spring AO-ENSO connection. Figure 1 displays scatterplots of the spring AO index with the subsequent winter Niño3.4 index, for those when the spring AO index and the preceding November AO index have the same and opposite signs. There are 17 (18) same (opposite) sign years over 1980–2014, including 1981, 1982, 1984, 1992, 1994, 1996, 1997, 1999, 2001, 2002, 2007, 2008, 2010, 2012, 2013, and 2014 (1980, 1983, 1986, 1987, 1989, 1990, 1991, 1993, 1995, 1998, 2000, 2003, 2004, 2005, 2006, 2009, and 2011). The correlation coefficient between the spring AO index and the following winter Niño3.4 index is as high as 0.65 for the same sign years, significant at the 99% confidence level (Fig. 1a). By contrast, the spring AO-ENSO connection is extremely weak during the opposite sign years, with the correlation coefficient being −0.01 (Fig. 1b). It should be mentioned that the results obtained from Fig. 1 are independent of the ENSO cycle (Supp. Fig. S5). Furthermore, the results remain the same when the near-zero November AO years (defined as those years when absolute values of the November AO index were less than 0.2 or 0.3) were excluded in constructing the scatterplots (Supp. Figs S6 and S7). These results indicate that the spring AO-ENSO relationship highly depends on the phase of the preceding November AO.

**Figure 2:**
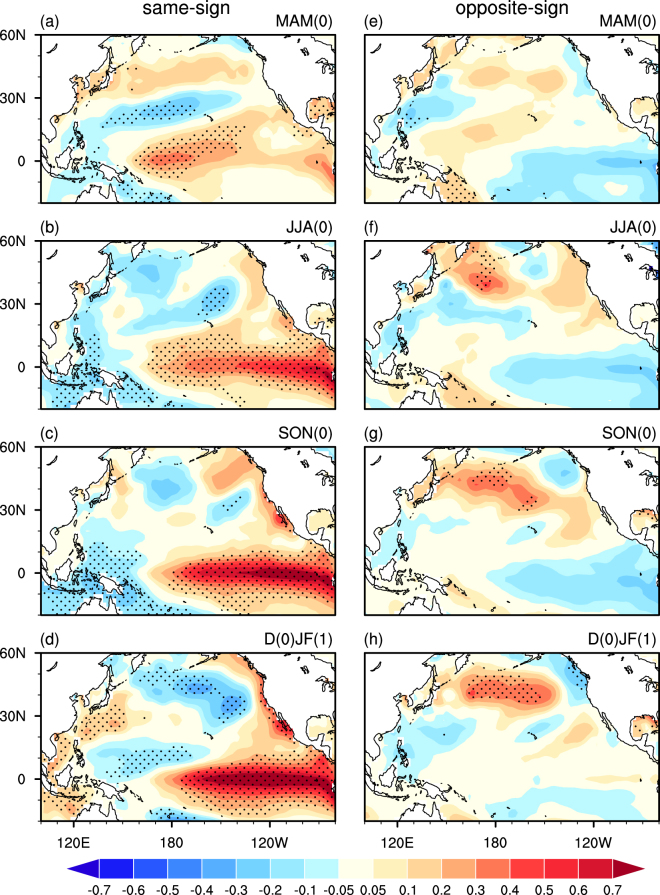
Anomalies of SST (°C) at (**a**,**e**) MAM(0), (**b**,**f**) JJA(0), (**c**,**g**) SON(0), and (**d**,**f**) D(0)JF(1) regressed upon the spring AO index, constructed respectively, for the years when the spring AO index and the preceding November AO index have the (left) same and (right) opposite signs. Stippling regions indicate SST anomalies that significantly difference from zero at the 95% confidence level.

**Figure 3:**
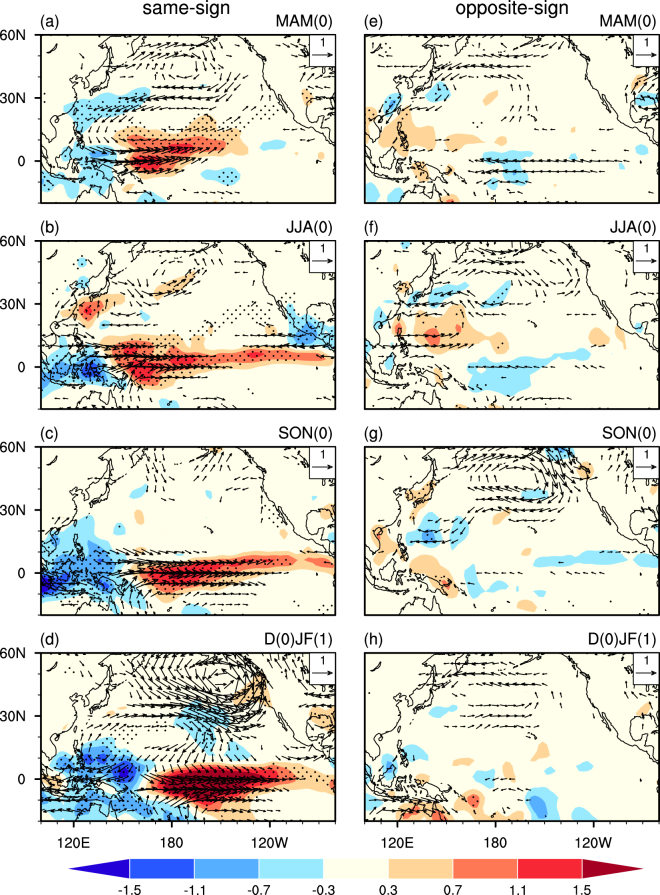
As in Fig. 2, but for 850 hPa winds (m s^−1^, vector) and precipitation anomalies (mm day^−1^, shading). Stippling regions indicate precipitation anomalies that significantly difference from zero at the 95% confidence level. Wind anomalies in both directions less than 0.2 m s^−1^ are not plotted.

Figure 2 compares the seasonal evolution of SST anomalies regressed upon the spring AO index between the years when the spring AO and the preceding November AO indices have the same and opposite signs. Figure 3 shows the related anomalies of 850hPa winds and precipitation. For the same sign years, the evolution of the SST, 850hPa wind and precipitation anomalies (Figs 2a–d and 3a–d) bears a close resemblance to those seen in Figs S2 and S3, but with larger amplitudes. In particular, similar to the physical process described above, a significant anticyclonic anomaly is apparent over the midlatitude North Pacific, accompanied by a pronounced cyclonic anomaly locating over the subtropical western-central Pacific in spring (Fig. 3a). The southwesterly wind anomalies over the subtropical central-western North Pacific lead to local SST warming and atmospheric heating (Figs 2a and 3a). The atmospheric heating anomalies, in turn, contribute to the generation of the westerly wind anomalies in the TWCP (Fig. 3a), leading to SST warming in the TCEP in the following summer via triggering an eastward propagating warm Kelvin wave (Fig. 2b). Finally, an El Niño-like SST warming is induced in the TCEP in the following winter via the Bjerknes positive air-sea feedback (Figs 2b–d and 3b–d).

By contrast, for the years when the preceding November AO and spring AO indices have the opposite sign, the spring AO-related cyclonic anomaly over the subtropical North Pacific is pretty weak in spring (Fig. 3e). As a result, the related SST warming and atmospheric heating anomalies are faded in the subtropics (Figs 2e and 3e). The TCEP is covered by weak cold SST anomalies from spring to winter (Figs 2e–h and 3e–h). Hence, there is no air-sea feedback to warm SSTs in the TCEP and no ENSO occurred. The comparison results demonstrate that November AO modulates the following spring AO-ENSO connection.

**Figure 4:**
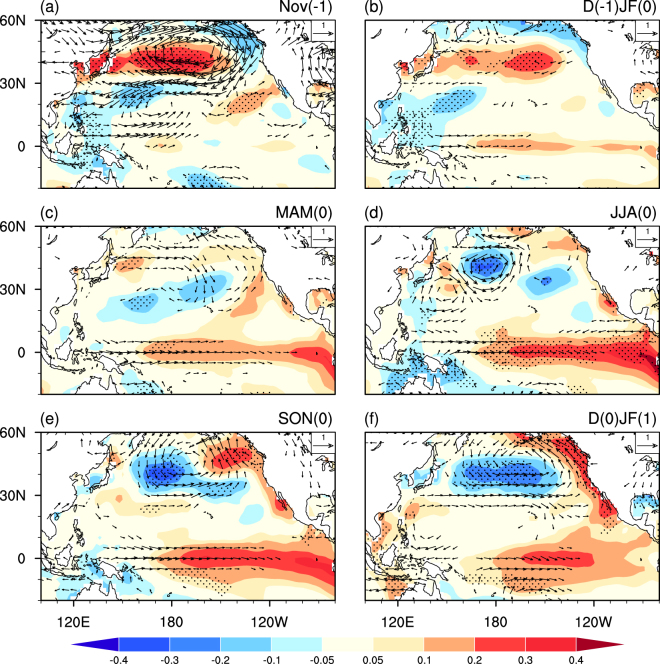
Anomalies of SST (°C, shading) and 850hPa winds (m s^−1^, vector) at (**a**) Nov(−1), (**b**) D(−1)JF(0), (**c**) MAM(0), (**d**) JJA(0), (**e**) SON(0), and (**f**) D(0)JF(1) regressed upon the normalized Nov(−1) AO index. Stippling regions indicate SST anomalies that significantly difference from zero at the 95% confidence level. Wind anomalies in both directions less than 0.2 m s^−1^ are not plotted.

What is the possible mechanism of the influence of November AO on the spring AO-ENSO connection? To address this question, we further show the November AO associated SST and 850hPa wind anomalies in Fig. 4. In the positive November AO years, a meridional atmospheric dipole pattern appears over the North Pacific. As detailed in Chen *et al*.^17^, pronounced cyclonic circulation and atmospheric heating anomalies are observed over the subtropical North Pacific. The atmospheric heating anomalies sustain westerly wind anomalies over the tropical western North Pacific through a Gill-like atmospheric response. The westerly wind anomalies extend eastward subsequently through positive air–sea feedback mechanism, and result in SST warming during the following spring and summer in the TCEP (Fig. 4b–f).

The correlation coefficient between November and the subsequent spring AO indices is only 0.1 (Supp. Fig. S1), indicating that the November AO associated SST and atmospheric anomalies are somehow independent of the spring AO associated counterparts. In the positive November AO years, the positive spring AO-induced warming anomalies in the tropical Pacific in spring and summer would be enhanced by the preceding positive November AO-induced SST anomalies (Fig. 4c,d). Hence an El Niño-like warming would be generated via the positive air-sea feedback (Fig. 3c,d). By contrast, when the preceding November AO is in a negative phase, the induced SST cooling anomalies in the TECP would reduce the spring AO-related SST warming anomalies in spring and summer, leading to weak cold SST anomalies in the TECP (Fig. 3e,f). As a result, there is no air-sea feedback to warm SSTs in the TCEP and thus no El Niño events would be generated in the following winter (Fig. 3g,h).

## Summary and Discussion

Previous studies have shown that the spring AO can exert a pronounced influence on the outbreak of ENSO events during the following winter. The present study further suggests that the spring AO-ENSO connection is significantly modulated by its preceding November AO condition. When the preceding November AO index is in phase with the spring AO index, the influence of the spring AO on ENSO is strong and significant. By contrast, when the November and spring AO indices are out of phase, there is no spring AO-ENSO connection. Therefore, the phase of the preceding November AO should be taken into account when applying the spring AO-ENSO relationship in the ENSO prediction.

The impact of the preceding November AO on the spring AO-ENSO relationship is mainly through the constructive and destructive superposition of the November and spring AO associated SST anomalies in the TCEP in spring and summer, as well as the SST anomalies developed further in the tropical Pacific in winter via the positive air-sea feedback. Nevertheless, it is noted that other processes may also exist for the modulation of the November AO on the following spring AO-ENSO connection, which need to be further explored.

The modulation of November AO on the spring AO-ENSO relationship has also been examined using a long time series of the AO index during 1899–2002 obtained from http://www.atmos.colostate.edu/~davet/ao/Data/ao_index.html (Supp. Figs S8 and S9). When November and spring AO indices have the same sign, the correlation coefficient between the spring AO and the subsequent winter Niño3.4 index is 0.42, significant at the 95% confidence level. By contrast, when they have the opposite sign, the correlation is only −0.01. This increases confidence in the results reported here based on the more reliable period from 1979 to 2015. In addition, an immediate question following the current study is: whether AO in other months (such as October and December) can also induce SST anomalies in the tropical Pacific and further modulate the spring AO-ENSO connection? Chen *et al*.^17^ has shown that only the AO variability in November and spring has a significant influence on the SST variation over the tropical central-eastern Pacific (please see their Figure 1). They demonstrated that the intensity of the North Pacific synoptic-scale eddy activity plays a crucial role in determining whether the AO can exert influences on the tropical Pacific SST anomalies. This is because the strength of the AO-generated atmospheric circulation anomalies over the subtropical North Pacific is positively related to the intensity of the North Pacific synoptic-scale eddy activity^17^. In addition, the annual cycle of the North Pacific synoptic-scale eddy activity shows a bimodal structure, with two maxima appearing in November and spring (please see their figure 10). Hence, it is suggested that only AO in November and spring can exert impacts on the tropical Pacific SST^17^.

## Electronic supplementary material

Supplementary Figures S1-S9
