# Supplementary material for: Modulation of the relationship between spring AO and the subsequent winter ENSO by the preceding November AO
Source: Sci Rep. 2018 May 2;8:6943. doi: 10.1038/s41598-018-25303-0 (PMC5932071; doi:10.1038/s41598-018-25303-0)
Supplement: Supplementary file 1 — Supplementary Figures S1-S9 [file 41598_2018_25303_MOESM1_ESM.pdf]

Supplementary Information for

"Modulation of the relationship between the spring AO and subsequent winter ENSO by the preceding November AO"

Shangfeng Chen[1], Wen Chen[1*], and Bin Yu[2]

[1]*Center for Monsoon System Research, Institute of Atmospheric Physics, Chinese Academy of Sciences, Beijing, China*

[2]*Climate Research Division, Environment and Climate Change Canada, Toronto, ON, Canada*

Corresponding author*:

Wen Chen

Institute of Atmospheric Physics

Chinese Academy of Sciences

Beijing 100029, China

e-mail: cw@post.iap.ac.cn

This file includes Supplementary Figures S1-S9, which can be described as follows:

Supplementary Figure S1 displays normalized time series of Nov(-1) AO index, spring (MA(0)) AO index, and following winter (D(0)JF(1)) Niño3.4 index.

Supplementary Figure S2 shows evolution of SST and 850hPa winds anomalies from spring to subsequent winter obtained by regression upon the normalized spring AO index.

Supplementary Figure S3 displays evolution of precipitation anomalies from spring to subsequent winter obtained by regression upon the normalized spring AO index.

Supplementary Figure S4 is similar to Supplementary Figure S2, but the D(-1)JF(0) Niño3.4 index has been linearly removed from the MA(0) AO index and the SST anomalous fields.

Supplementary Figure S5 is similar to Figure 1 (in the main manuscript), but the D(-1)JF(0) Niño3.4 index has been linearly removed from the D(0)JF(1) Niño3.4 index and MA(0) AO index

Supplementary Figure S6 is similar to Figure 1 (in the main manuscript), but those years were rejected in constructing this figure when absolute values of the November AO index were less than 0.2.

Supplementary Figure S7 is similar to supplementary Figure S6, but those years were rejected in constructing this figure when absolute values of the November AO index were less than 0.3.

Supplementary Figure S8 is similar to Figure 1 (in the main manuscript), but based on the AO index during 1899-2002 obtained from http://www.atmos.colostate.edu/~davet/ao/Data/ao_index.html.

Supplementary Figure S9 is similar to Figure 2 (in the main manuscript), but based on the data during 1899-2002.

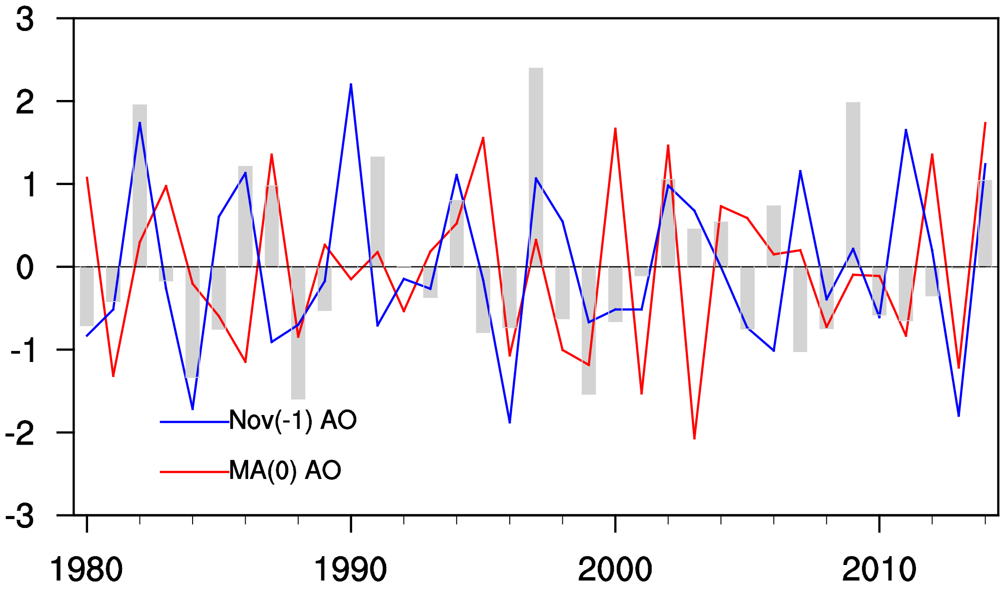

Figure S1. Normalized time series of Nov(-1) AO index (blue line), spring (MA(0)) AO index (red line), and following winter (D(0)JF(1)) Niño3.4 index (bar).

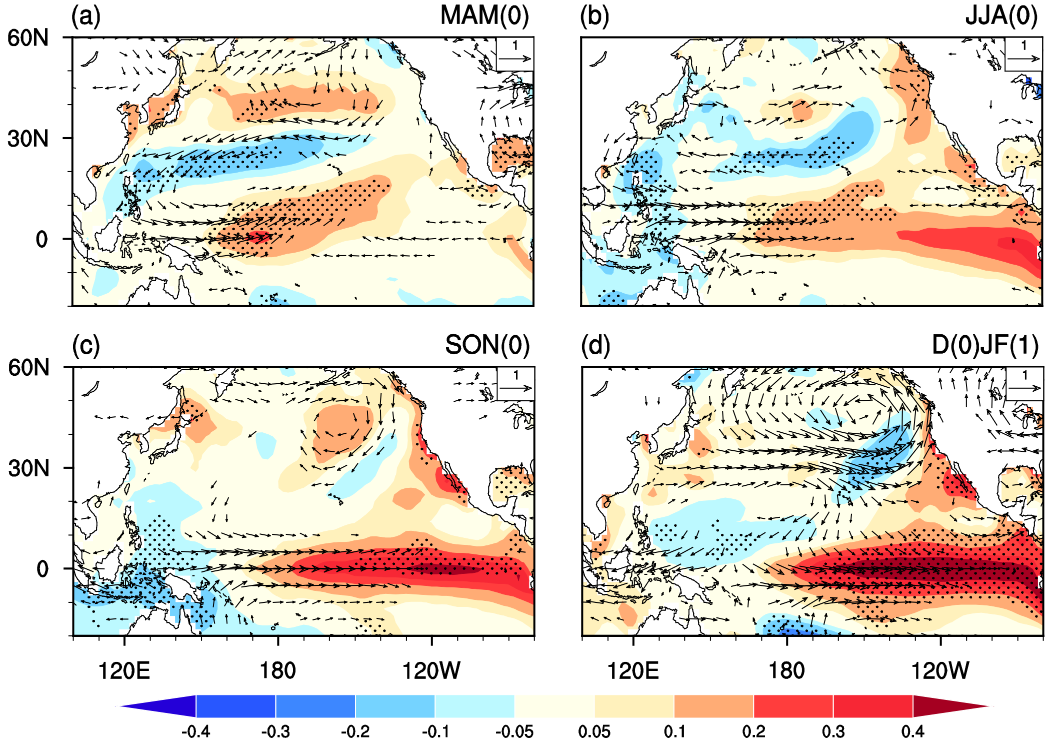

Figure S2. Anomalies of SST (°C, shading) and 850hPa winds (m s^{-1}, vector) at (a) MAM(0), (b) JJA(0), (c) SON(0), and (d) D(0)JF(1) regressed upon the normalized spring AO index. Stippling regions indicate SST anomalies that significantly difference from zero at the 95% confidence level.Windanomalies in both directions less than 0.2 m s^{-1} are not plotted.

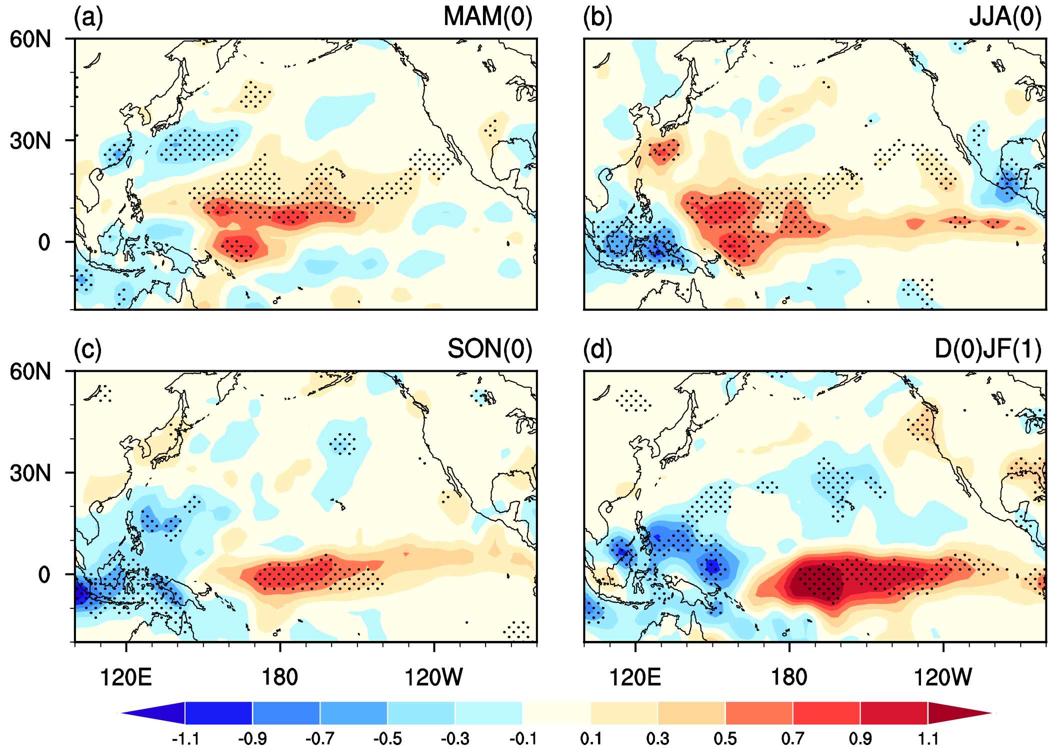

Figure S3. As in Fig.S2, but for precipitation anomalies regressed upon the spring AO index (mm day^{-1}). Stippling regions indicate SST anomalies that significantly difference from zero at the 95% confidence level.

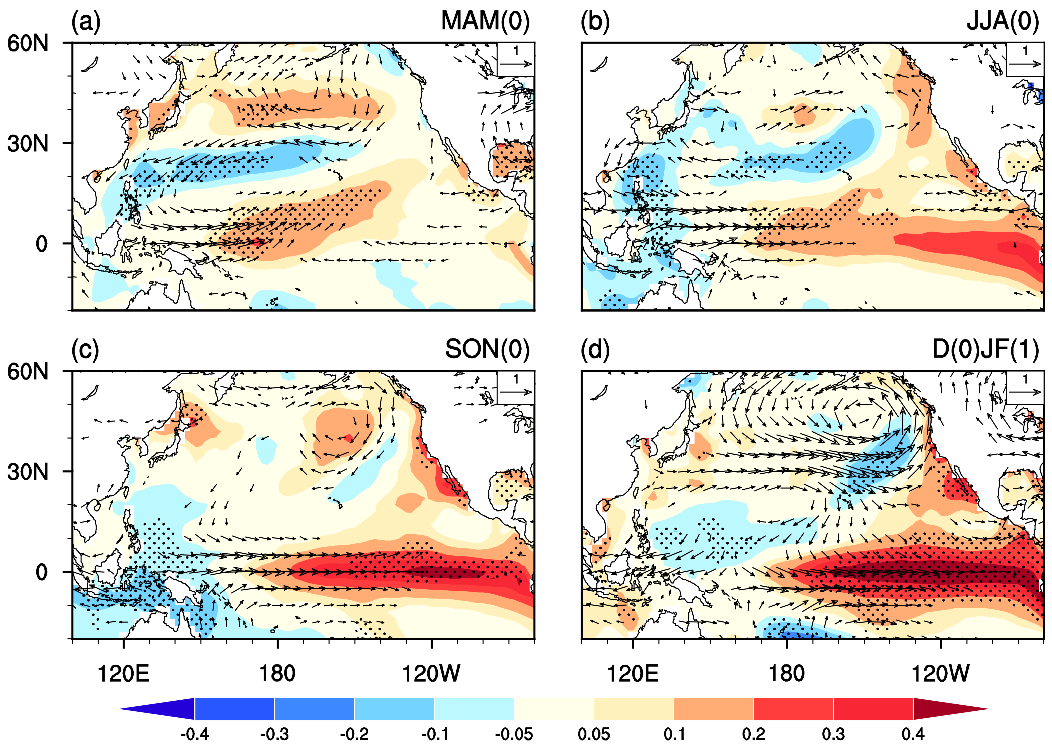

Figure S4. As in Fig. S2, but the D(-1)JF(0) Niño3.4 index has been linearly removed from the MA(0) AO index and the SST anomalous fields.

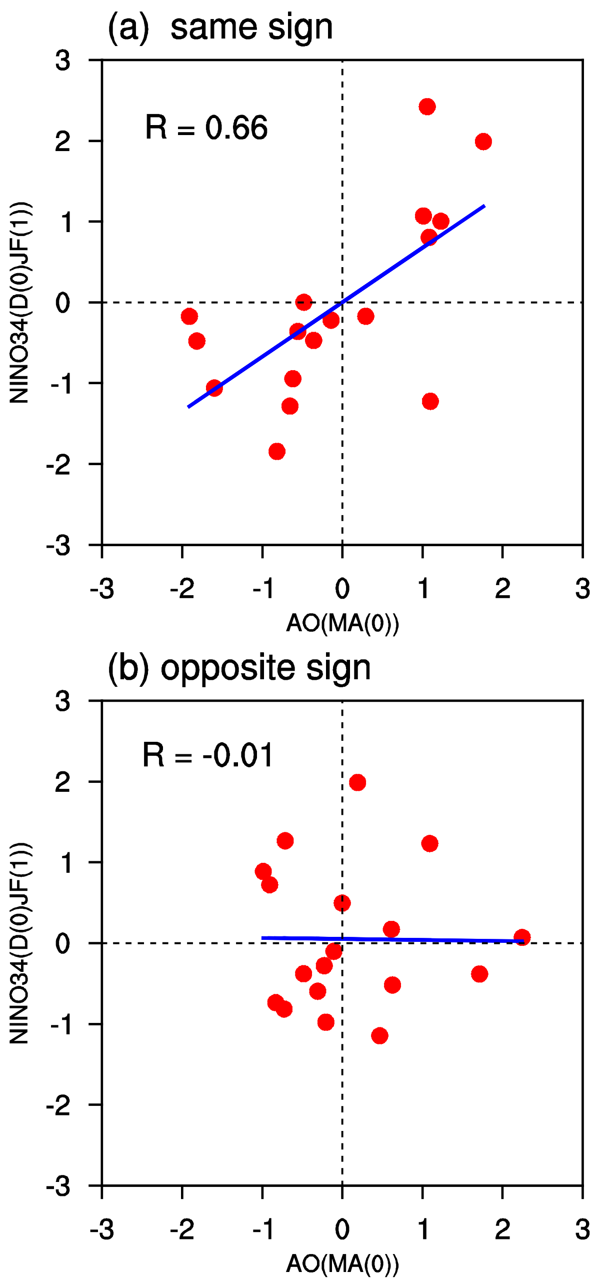

Figure S5. Scatterplots of the spring AO index with the subsequent winter Niño3.4 index, for the years when the spring AO index and the preceding November AO index have the (a) same and (b) opposite signs, respectively. It is noted that the D(-1)JF(0) Niño3.4 index has been linearly removed from the D(0)JF(1) Niño3.4 index and MA(0) AO index when constructing this figure.

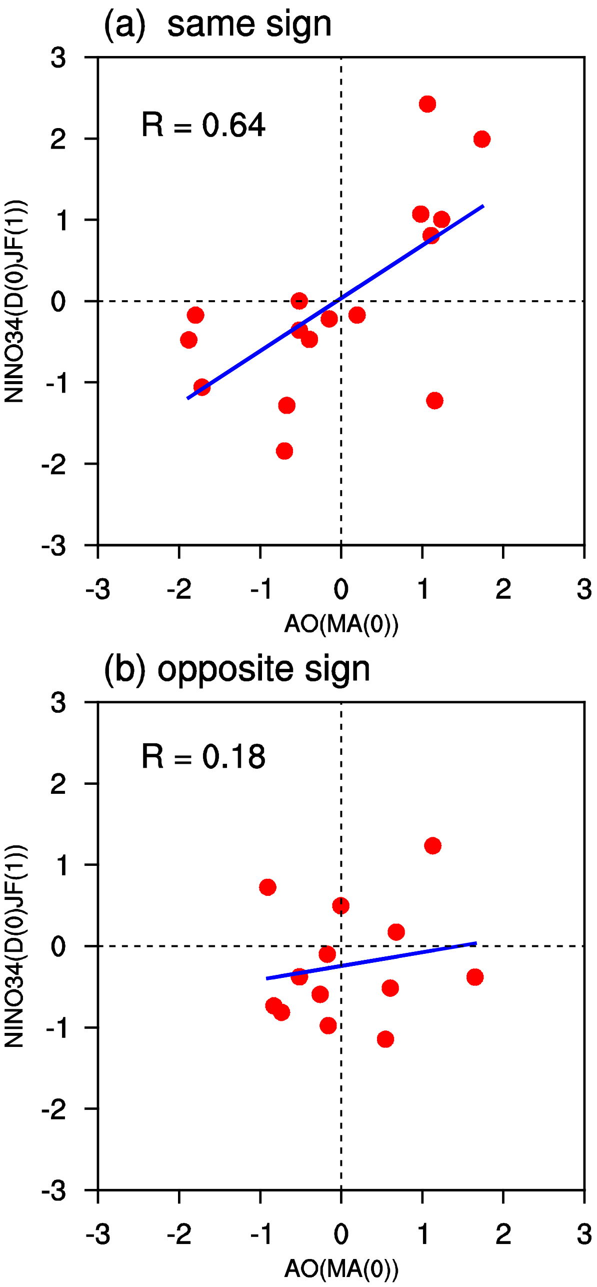

Figure S6. As in Fig. 1 in the main manuscript, but those years were rejected in constructing this figure when absolute values of the November AO index were less than 0.2.

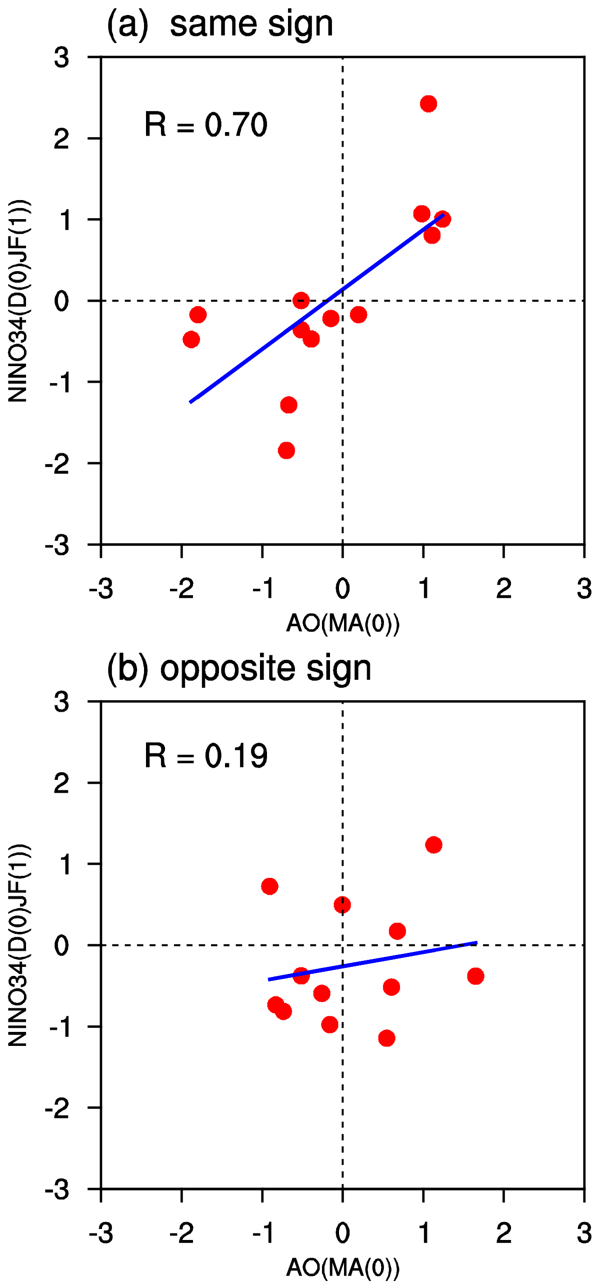

Figure S7. As in Fig. S6, but those years were rejected in constructing this figure when absolute values of the November AO index were less than 0.3.

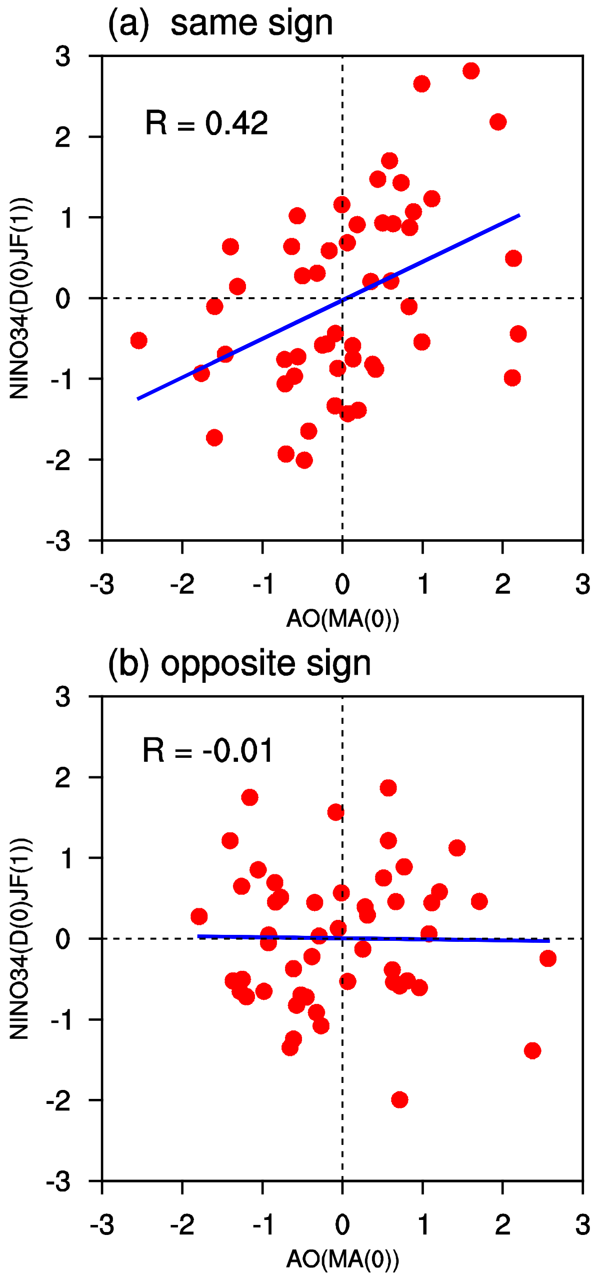

Figure S8. As in Figure 1 in the main manuscript, but based on the AO index during 1899-2002 obtained from http://www.atmos.colostate.edu/~davet/ao/Data/ao_index.html.

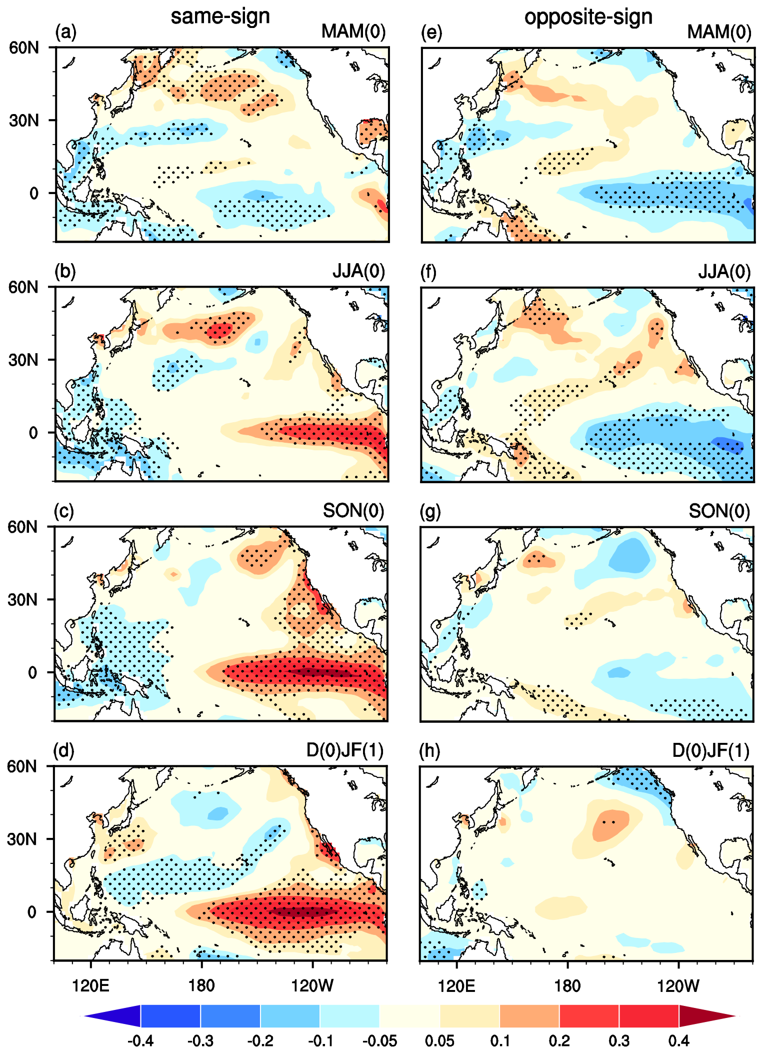

Figure S9. As in Figure 2 in the main manuscript, but based on the data during 1899-2002.
